# A Geometrical Study on the Roof Tile-Shaped Modes in AlN-Based Piezoelectric Microcantilevers as Viscosity–Density Sensors

**DOI:** 10.3390/s19030658

**Published:** 2019-02-06

**Authors:** Víctor Ruiz-Díez, Javier Toledo, Jorge Hernando-García, Abdallah Ababneh, Helmut Seidel, José Luis Sánchez-Rojas

**Affiliations:** 1 Microsystems, Actuators and Sensors Group, Universidad de Castilla-La Mancha, 13071 Ciudad Real, Spain; javier.toledo.serrano@gmail.com (J.T.); Jorge.Hernando@uclm.es (J.H.-G.); JoseLuis.SAldavero@uclm.es (J.L.S.-R.); 2 Electronic Engineering Department, Hijjawi Faculty for Engineering Technology, Yarmouk University, 21163 Irbid, Jordan; a.ababneh@lmm.uni-saarland.de; 3 Chair of Micromechanics, Microfluidics/Microactuators, Faculty of Natural Sciences and Technology II, Saarland University, 66123 Saarbrücken, Germany; seidel@LMM.Uni-saarland.de

**Keywords:** high Q-factor, liquid media, out-of-plane, piezoelectric, AlN, cantilever

## Abstract

Cantilever resonators based on the roof tile-shaped modes have recently demonstrated their suitability for liquid media monitoring applications. The early studies have shown that certain combinations of dimensions and order of the mode can maximize the Q-factor, what might suggest a competition between two mechanisms of losses with different geometrical dependence. To provide more insight, a comprehensive study of the Q-factor and the resonant frequency of these modes in microcantilever resonators with lengths and widths between 250 and 3000 µm and thicknesses between 10 and 60 µm is presented. These modes can be efficiently excited by a thin piezoelectric AlN film and a properly designed top electrode layout. The electrical and optical characterization of the resonators are performed in liquid media and then their performance is evaluated in terms of quality factor and resonant frequency. A quality factor as high as 140 was measured in isopropanol for a 1000 × 900 × 10 µm^3^ cantilever oscillating in the 11th order roof tile-shaped mode at 4 MHz; density and viscosity resolutions of 10^−6^ g/mL and 10^−4^ mPa·s, respectively are estimated for a geometrically optimized cantilever resonating below 1 MHz.

## 1. Introduction

The use of miniaturized resonant devices to monitor liquid properties is of interest to many different fields, such as automotive industry, biology or food analysis [1,2]. Microcantilever resonator-based rheological sensors have demonstrated the advantages of miniaturization over traditional macroscopic setups, such as a reduced sample volume (~picoliter) and fast measurement times (~seconds), at resolutions that can reach 10^−5^ g/mL and $10^{-3}$ mPa·s, for the density and viscosity respectively [3,4,5]. Applications in which a small concentration of a solute needs to be detected in a liquid medium [6] take advantage of a high resolution in the determination of its rheological properties, so for a cantilever sensor-based setup, an appropriate design of the resonator and convenient vibrational mode might be necessary. For such liquid media applications, a vibration mode with reduced energy losses is crucial for a good signal to noise ratio and accurate resolution of the sensor. Among the different modes of vibration, it is already well established to use in-plane modes for an efficient performance in liquid media [7,8]. On the other hand, out-of-plane modes are considered to yield lower quality factors and may be less suitable for such application scenarios [9,10]. However, especially the roof-tile shaped modes have recently demonstrated their suitability for liquid media applications, due to high quality factors and reasonably low resonant frequencies [11,12]. Preliminary studies involved the characterization in isopropanol of the first order of this mode [11], with a Q-factor value around 35, and of higher orders [13], with Q-factors above 200. The optimization of these modes and their application as viscosity and density sensors has recently been object of different studies [6,14,15], exhibiting density resolutions as low as $1.5\times 10^{-7}$ g/mL and viscosity resolutions as low as $1.3\times 10^{-4}$ mPa·s. Those preliminary studies established the basic methodology and calibration procedure, while showing the potential in the use of a particular roof tile-shape mode. However, a geometrical study of their outstanding in-liquid behavior has not been fully addressed.

In the present work, we focus on the analysis of the fluid-structure interaction when exciting one-sided clamped MEMS resonators in the roof tile-shaped modes. These modes can also be labelled as 1n-modes according to Leissa’s nomenclature [16,17] for plates, where n is the number of nodal lines parallel to the length of the cantilever and *1* represents the nodal line at the support of the cantilever. The performance of the devices under study will be evaluated in terms of quality factor and resonant frequency by means of the electrical impedance of the integrated piezoelectric layer.

## 2. Materials and Methods

Micromachined cantilevers, with lengths varying from 250 to 3000 µm and widths in the range of 300 to 1500 µm, were designed and fabricated in four different thicknesses (i.e., 10, 20, 40, and 60 µm) to study the in-liquid behavior of the roof tile-shaped mode. Two different fabrication processes were employed, depending on the device thickness.

**Figure 1:**
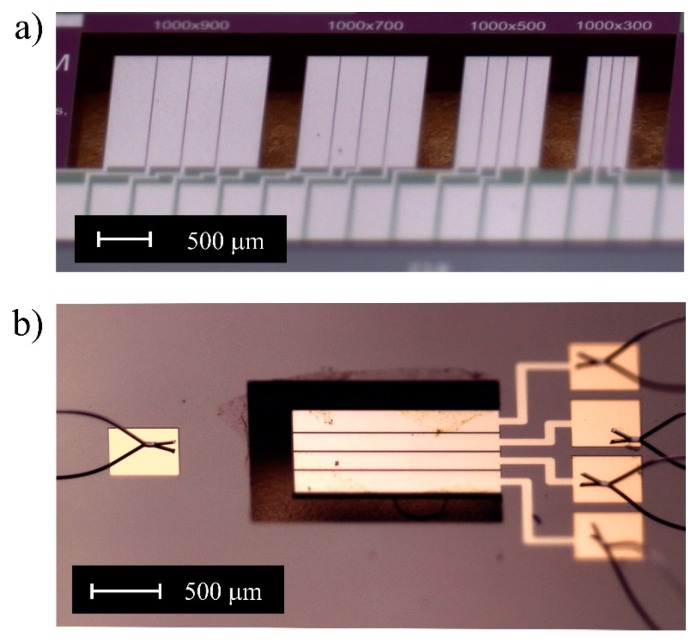
Optical micrograph of (**a**) a set of 1000 µm-length and 10 µm-thick cantilevers with different widths from 300 to 900 µm and (**b**) a 40 µm thick cantilever with a length of 1500 µm and a width of 700 µm. The top electrode is split in four stripes that can be accessed individually with respect to the common bottom electrode. All of them are wire bonded to a standard dual in-line package (DIP).

The fabrication of the thinnest cantilevers, such as the ones depicted in Figure 1a, was carried out by MEMSCAP, following the PiezoMUMPS foundry process [18]: a SOI wafer with 10 μm thick device layer is covered with a 500 nm thick aluminum nitride piezoelectric film. The silicon is doped to serve as both bottom electrode and structural layer. As top electrode, a stack of chrome (20 nm) and aluminum (1 μm) is deposited.

The fabrication of the rest of devices (see Figure 1b) followed a different process: a p-doped (100) silicon plate—wet etched to a thickness of 20, 40, or 60 µm—serves as bottom electrode, which is covered with a 1 μm thick AlN piezoelectric film synthesized in a reactive sputter process from an aluminum target in pure nitrogen atmosphere. As top electrode, a stack of chromium (20 nm) and gold (0.5 μm) is applied. A complete description of the fabrication process can be found in [19].

**Figure 2:**
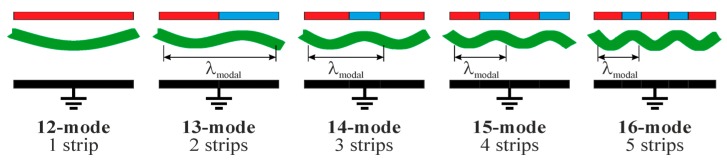
Schematic of the modal shapes and sign (red and blue for positive and negative applied voltage) of electrode potential in cross sectional view. The modal wavelength ($\lambda_{modal}$) is also indicated.

A striped pattern was used as top electrode layout, with a common bottom electrode, which allowed to measure the different polarization charges generated locally due to the bending characteristics of the mode shape. An efficient actuation of the different roof tile-shaped modes was made possible by a proper polarization of the right stripes [20], as it can be seen in Figure 2. As an example, for an optimal actuation of the 15-mode, electrode pairs 1 and 3 should be short-circuited and subjected to a positive potential while electrode pairs 2 and 4 should be subjected to a negative potential, with respect to the common bottom electrode. Doing so, the total charge collected by the electrodes is maximized in comparison to the same device with a non-striped electrode configuration [12].

**Figure 3:**
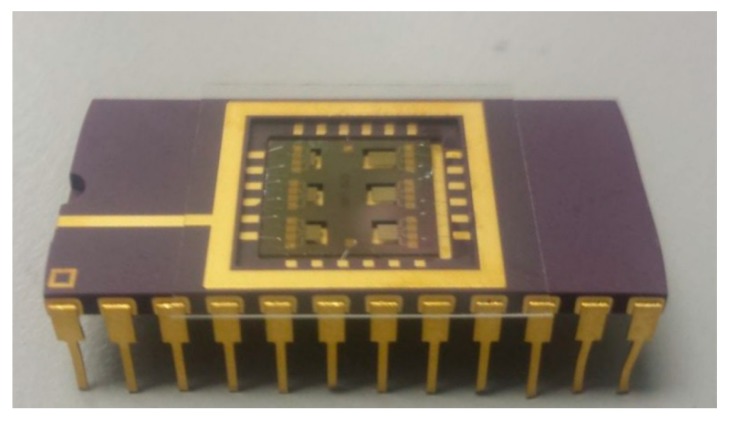
Photograph of a sensor die containing six cantilever resonators. The die was glued and bonded inside the cavity of the package, which was filled with liquid and covered with a thin glass for the experiments.

The corresponding dies were glued and wire-bonded in 24-pin DIP packages, whose cavity serve as fluid cell when closed by a thin cover glass on the top (see Figure 3). A small quantity of fluid (~100 µL) was placed inside this cell and the measurements were taken at room temperature (25 ± 1 °C).

**Figure 4:**
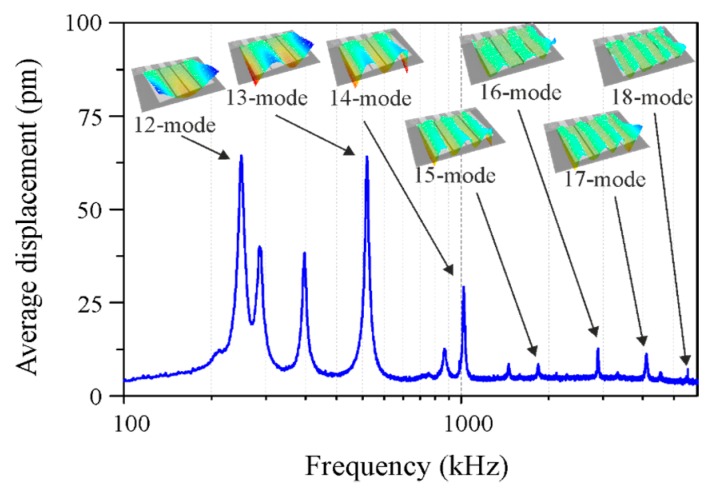
Average displacement per unit voltage from the optical characterization of the 500 × 700 × 20 µm^3^ cantilever in isopropanol. The measured modal shapes are shown next to each peak.

The in-liquid performance of the 1n-modes in the cantilevers under study was assessed optically and electrically. A scanning laser Doppler vibrometer (MSV 400 Polytec) was used for the optical characterization. This instrument provides a laser spot which can scan a grid of points on the top plate surface to measure the out-of-plane component of either velocity or displacement. Therefore, an identification of all the out-of-plane modes of any structure can easily be done with this equipment. Figure 4 shows the displacement spectrum for a MEMS resonator (500 × 700 × 20 µm^3^) in isopropanol, together with the recorded modal shapes up to the 18-mode. As it can be seen in the spectrum example, other modes were detected in the optical characterization but their performance in terms of Q-factor in different liquids and the other dimensions considered, were not comparable to the roof tile-shape modes. Figure 4 shows a second order flexural mode peak at 255 kHz and a second order torsional mode peak at 346 kHz, but their response was weaker than the surrounding roof tile-shaped and their Q-factor lower, making them less suitable for in-liquid sensing. In addition, those mode families were not optically detected in thicker devices, because of the greater fluid damping.

**Figure 5:**
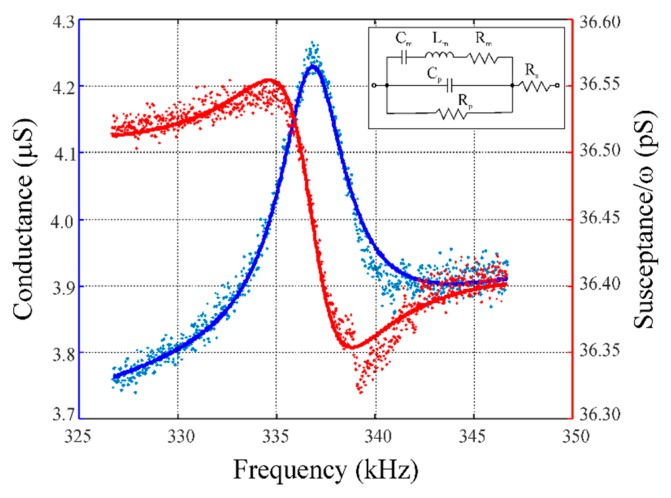
Electric impedance spectrum (dotted line) for a 1500 × 900 × 40 µm^3^ cantilever near the first order roof tile-shaped mode frequency in isopropanol. The peak represents the motional response of mode under study and from the fitting to the circuit model (solid line) the figures of merit of the resonance can be deduced. Inset: modified Butterworth–Van-Dyke equivalent electrical circuit for a piezoelectric resonator.

Thanks to the integrated piezoelectric layer, an all-electrical actuation/detection scheme is possible and hence, the electrical performance of the AlN-actuated devices could be studied by recording the impedance spectrum of the different modes of vibration. For this purpose, a 4294A Agilent impedance analyzer was used. The obtained impedance spectrum is then fitted to a modified Butterworth–Van-Dyke equivalent circuit model (see Figure 5), in order to obtain the equivalent electrical parameters of motion [21]. R_p_ and C_p_ represent the parasitic resistance and capacitance related to the piezoelectric film and the R_m_-L_m_-C_m_ arm represents the motional behavior of the structure. An additional resistance R_s_ was added to model the electric paths and bonding resistances. The resonant frequency of each mode and other figures of merit, such as the quality factor or the motional resistance, can be derived from these electrical parameters.

## 3. Results and Discussion

In the following section, the more relevant results from the comprehensive geometrical study of the in-liquid behavior of the roof tile-shaped modes are presented. All the devices were optically and electrically characterized while fully immersed in isopropanol (i.e., density of $\rho =781.2\;\mathrm{kg}/m^{3},$ viscosity of η=2.1062 mPa·s, and speed of sound of c=1139 m/s).

**Figure 6:**
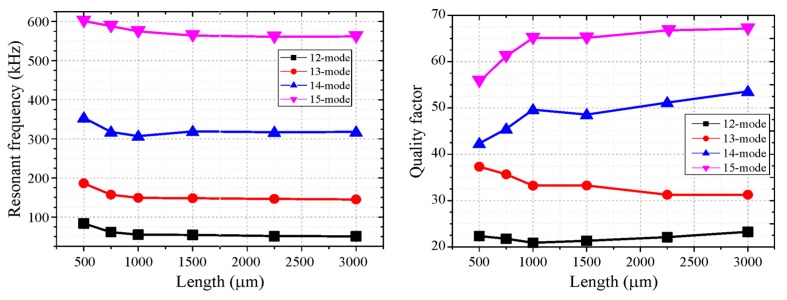
Resonant frequencies and quality factors for the first four roof tile-shaped modes in cantilevers with different lengths, and constant width and thickness of 900 µm and 20 µm, respectively. Values were deduced from the electrical impedance measurements in isopropanol.

The resonant frequency in vacuum for the roof tile-shape modes in slender beams depends on the thickness and width of the cantilever [11], and on the mechanical properties of the structure. In this regard, the length should have no practical influence on the resonant frequency, if the structure can be considered as slender (width far less than length). Figure 6 shows the evolution of the quality factor and resonant frequency for the first four roof tile-shaped modes in isopropanol in 900 µm-wide, 20 µm-thick cantilevers of lengths from 500 to 3000 µm. As we can see, decreasing the length has no practical effect into the resonant frequency, as long as the length to width ratio is above unit. For lower ratios, the resonant frequency tends to increase due to the influence of the anchoring, being more relevant for higher orders of the roof tile-shaped mode. Regarding the quality factor, differences below 5% of the mean value were observed for lengths above the cantilever width, while for low length-to-width ratios, the maximum difference was about 15% of the value. This can be attributed to the change in the resonance frequency previously noted, more than to a change in the mechanism of losses.

**Figure 7:**
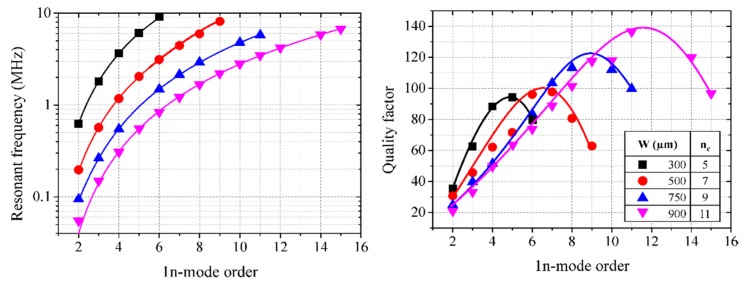
Resonant frequencies and quality factors as a function of order of the roof tile-shaped mode for the 1000 µm long, 10 µm thick cantilevers, deduced from the electrical impedance measurements in isopropanol. The coincidence point (*n_c_*) is also given for each width.

The effect of the cantilever width on the in-liquid behavior of the roof tile-shaped modes was studied before for thin cantilevers [20]. For a given width, as the order of the mode increased, a maximum in the quality factor could be found for a particular order. This behavior is illustrated here in Figure 7 for 1000 µm-long, 10 µm-thick cantilevers.

This type of behavior, which was found in other kinds of modes was attributed to the appearance of acoustic losses, in addition to the already present viscous losses [8,22]. The compressibility of the medium is often neglected for micrometer sized structures at low order modes, where the spatial period of the modal shape, $\lambda_{modal}$ (see Figure 2) is much smaller than the wavelength of the acoustic oscillations of the surrounding medium, $\lambda_{accoustic}$. Therefore, as the mode number increases, the acoustic and modal wavelengths might become comparable and the mode number at which this happens is called coincidence point $n_{c}$ [20]

(1) $$n_{c}≅\frac{2c}{\pi }\sqrt{\frac{3\rho_{s}\left(1-\nu_{s}^{2}\right)}{E_{s}}}\left(\frac{f_{n}^{vac}}{f_{n}^{liq}}\right)\frac{W}{T},$$

where c is the speed of sound in the fluid, $\rho_{s}=2329\;\mathrm{kg}/m^{3},\;E_{s}=169\mathrm{GPa},\;\nu_{s}=0.30$ are the structure’s density, Young’s modulus, and Poisson ratio, $f_{n}^{vac},\;f_{n}^{liq}$ are the resonant frequencies in vacuum and liquid, and W,T are the cantilever width and thickness, respectively.

The coincidence point can be estimated using the above approximation and, as shown in Figure 7, there is a good agreement between the calculated coincidence point and the mode with the maximum quality factor. For modes below the coincidence point, decreasing the cantilever width leads to an increase in the quality factor, due to a higher resonant frequency. However, a narrow cantilever might exhibit the coincidence point, and hence the maximum in the quality factor, at a quite low order of mode, not being able to reach higher values. On the contrary, increasing the cantilever width, rises the coincidence point, what allows reaching higher orders of the roof tile-shaped mode before the acoustic losses become relevant.

**Figure 8:**
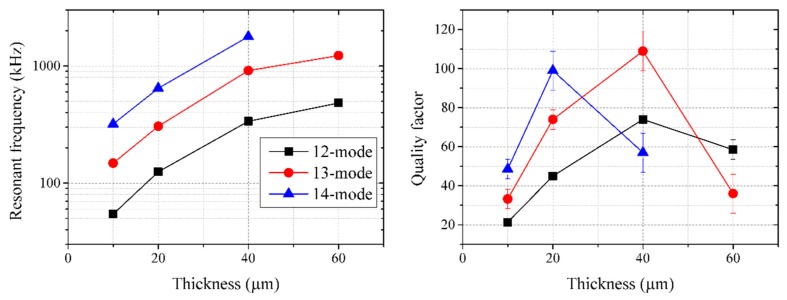
Resonant frequencies and quality factors for the first three roof tile-shaped modes in cantilevers with different thickness and constant length and width of 1500 µm and 900 µm, respectively.

Finally, the effect of the device thickness was studied. Figure 8 shows the resonant frequencies and quality factors for the first three roof tile-shaped modes in 1500 µm-long and 900 µm-wide cantilevers. Regarding the resonant frequency, increasing the thickness leads to an increase of the resonant frequency since the resonator mechanical stiffness is increased. With respect to the quality factor, for each mode, there is an increase of the quality factor as the thickness increases from low values. This is due a higher resonator mass, hence more energy stored, while only the viscous losses are present. However, increasing further the device thickness and, consequently, the resonant frequency, may decrease the acoustic wavelength to the point that it becomes comparable to the modal wavelength, leading to the appearance of the acoustic losses, and therefore the decrease of the quality factor.

As it can be seen from Figure 8, for each roof tile-shaped mode, there is a particular thickness at which the quality factor reaches its maximum value, and, as the order of the mode increases, the optimum thickness is decreased. In other words, for a given resonator thickness, there is an optimum roof tile-shaped mode that maximizes the quality factor, which is related to the respective mode of the coincidence point. For example, the 40 µm-thick cantilever exhibited a Q-factor of 110 when resonating in the 13-mode, but increasing the order to the 14-mode, halved the Q-factor to 55.

**Figure 9:**
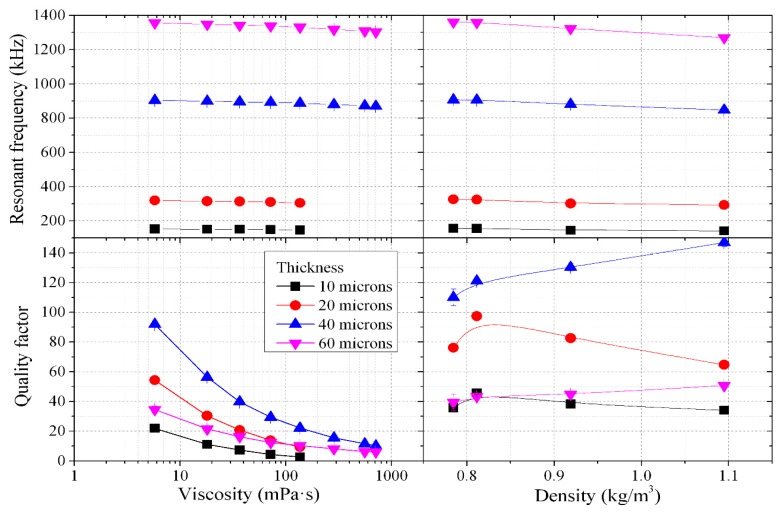
Resonant frequencies and quality factors for the second order roof tile-shaped mode (13-mode) in 1500 µm-long, 900 µm-wide cantilever devices of four different thicknesses in a wide range of liquid media. The left graphs show, as a function of the liquid viscosity, the experimental results in liquids with similar density (between 0.838 and 0.872 g/mL) while right graphs show, as a function of the liquid density, the results in liquids with similar viscosity (between 1.45 and 2.29 mPa·s).

In order to study the impact of such quality factor in a viscosity-density sensor based on the roof tile-shaped mode, the same devices with a constant length and width of 1500 and 900 µm, respectively, were studied in media with different viscosities (from 0.4 to 713 mPa·s) and densities (from 0.684 to 1.49 g/mL), chosen from calibration standards and common solvents, implying fixed combinations of viscosity and density measurement points. Figure 9 presents the evolution of the quality factor and the resonant frequency of the 13-mode, for four different thicknesses with the viscosity and density. For the sake of clarity, the values of the resonance frequency and the Q-factor versus the viscosity are only shown for a set of liquids with very similar density, ranging from 0.838 to 0.872 g/mL. While for the evolution of these figures of merit with the density, only liquids similar in viscosity are considered, from 1.45 to 2.29 mPa·s.

The devices showed the typical interdependence of the figures of merit ($f_{n}^{liq},Q$) with the rheological properties of the liquids for an out-of-plane mode [23]: the quality factor is more sensitive to changes in the viscosity than to those in the density, with the opposite trend for the frequency. It can be seen in Figure 9 that the effect of viscosity change on the quality factor depends on the resonator thickness. As it was seen in Figure 8, for a viscosity around 2.5 mPa·s, the optimum thickness corresponds to a value of 40 µm. However, as the viscosity increases, the viscous losses increase, while the acoustic losses remain invariant. So, in cantilever devices with a thickness value below the optimum, the Q-factor is dominated by the viscous losses and a change in the viscosity would translate into a decrease of the Q-factor. At the same time, an increase in the viscosity would displace the optimum thickness to a larger value. This can be observed in Figure 9, where the decrease rate of the Q-factor with the viscosity is much lower for 60 µm thickness—which is above optimum at low viscosities—than for the others. Therefore, the best geometry in terms of Q-factor would depend on the properties of the medium, since a viscosity, density or speed of sound change might alter the trade-off between the acoustic and the viscous losses.

The set of measurements in different liquids allows the calibration of the sensor and its further application to different media within the calibration range. One possible calibration method consists in the decomposition of the figures of merit, Q-factor and resonant frequency in terms of the resonant frequency in vacuum $f_{n}^{vac}$, the modal mass m, and two parameters associated with the fluid-structure interaction, i.e. the damping coefficient $g_{1}$ and added mass $g_{2}$ [23,24].

(2) $$f_{n}^{liq}=\frac{f_{n}^{vac}\sqrt{1-\frac{1}{2Q^{2}}}}{\sqrt{1+\frac{g_{2}}{m}}},$$

(3) $$Q=\frac{2\pi f_{n}^{vac}\sqrt{1+\frac{g_{2}}{m}}}{\frac{g_{1}}{m}}.$$

The parameters $g_{1}$ and $g_{2}$ are defined assuming that the force of the fluid on the plate in each point and direction can be decomposed into a dissipative term proportional to the velocity and an inertial part proportional to the acceleration, being *g*_1_ and *g*_2_ the respective proportionality constants. Their value depends on the fluid properties and on the geometry of the device and modal shape. In absence of an analytical model for the complete fluid-structure interaction of these modal shapes, the dependence of the damping coefficient and added mass with the viscosity, η and density, ρ may be estimated using a Taylor series expansion with four coefficients $C_{1}-C_{4}$ [15,23,25]:

(4) $$g_{1}\left(f_{n}^{liq}\right)=C_{1}\sqrt{f_{n}^{liq}}\sqrt{\eta \rho }+C_{2}\eta ,$$

(5) $$g_{2}\left(f_{n}^{liq}\right)=C_{3}\rho +\frac{C_{4}}{\sqrt{f_{n}^{liq}}}\sqrt{\eta \rho }.$$

This four-term Taylor expansion allows for the independent determination of the viscosity and density without the need of independent variation of both properties, thanks to the coefficients C_2_ and C_3_, which are normally zero in mode shapes with in-plane interaction [23].

The estimated coefficients for the devices studied in Figure 9 are shown in Table 1, together with the mean error, using this calibration expansion in the determination of the viscosity and density of the liquids previously measured. As it can be seen from Table 1, the devices exhibit a lower calibration error in the determination of the density as compared to the error of the viscosity, which is partly due to the higher range of values in the latter. It is also noticeable that the lower calibration errors were obtained in the 40 µm-thick structures, followed closely by the 60 µm-thick device instead of the 20 µm-thick one. This can be understood with the previously explained evolution of the Q-factor with the viscosity. The mean calibration error in density and viscosity might be improved by choosing an appropriate set of liquids for the calibration as well as increasing the number of constants of the series expansion [6].

Finally, using the above calibration coefficients and by uncertainty propagation from the resonant frequency and Q-factor to density and viscosity, the resolution in viscosity and density for the roof tile-shaped based sensors could be estimated. To complete this, the uncertainty in the determination of the resonant frequency and quality factor should be registered, once the electronic circuit accompanying the sensor is assessed. In order to give an estimation of these uncertainties without building the electronic circuitry, we make use of cantilever structures with similar dimensions, resonating in the fourth order of the roof tile-shaped mode, that were studied previously [15], where a relationship between them and the Q-factor was demonstrated. For a device like the 1500 × 900 × 40 µm^3^ cantilever, with the highest Q-factor value in the measured range of viscosities and densities, the 13-mode based sensor would have resolutions in density between 6.0 × 10^−7^ and 9.7 × 10^−5^ g/mL, for densities between 0.684 and 1.49 g/mL, respectively and resolutions in viscosity between 4.6 × 10^−4^ and 0.59 mPa·s, for viscosities between 0.4 and 713 mPa·s.

## 4. Conclusions

The roof tile-shaped modes studied in the present work showed quality factors that, in most of the geometries, surpass a value of 100, for resonant frequencies in the megahertz range. The geometrical study was performed experimentally, considering the length, width, and thickness of the cantilever structures. The effect of the acoustic losses on micron-sized resonators, already described in the literature for the roof tile-shaped modes, was also found in the current fabricated cantilevers. The effect of different geometric dimensions on the device performance were studied, revealing that for a given cantilever width or thickness, a maximum quality factor is obtained for that roof tile-shaped mode where acoustic and viscous losses become equal. The order of this optimum mode, or coincidence point, can be estimated according to the ratio of the acoustic vibration wavelength and the modal wavelength in the structure. It was demonstrated that for higher coincidence points a higher maximum Q-factor can be reached. This might suggest that any reduction of the acoustic losses should lead to even higher Q-factors. In addition, the effect of the cantilever length was found negligible on both Q-factor and resonant frequencies for the slender cantilevers, leaving a free parameter that might be used for optimizing other figures of merit such as the electrical response, which is mainly related to the total resonator area.

Finally, measurements in different media were carried out with cantilever devices of different thicknesses. Using a well-stablished calibration procedure, the applicability of some of the best resonators in this study was investigated. The most recent works in the field of microcantilever-based rheological sensors reported resolutions for the density and viscosity of 10^−5^ g/mL and 10^−3^ mPa·s, respectively. Those values were already surpassed by an early study using a non-optimized roof tile-shaped mode, exhibiting resolutions of 10^−7^ g/mL in a range of densities from 0.832 to 0.872 g/mL and 10^−4^ mPa·s in a range of viscosities from 5 to 700 mPa·s. Here, with a 1500 × 900 × 40 µm^3^ cantilever operating below 1 MHz, in the second order roof tile-shaped mode, density resolutions of 10^−6^ g/mL and viscosity resolutions of 10^−4^ mPa·s were estimated in an extended range of both density (from 0.684 to 1.49 g/mL) and viscosity (from 0.4 to 713 mPa·s). The geometrical study confirmed that an inappropriate selection of the resonator can have a detrimental effect on the Q-factor, and consequently on the performance of the cantilever as a viscosity and density sensor.

## Figures and Tables

**Table 1:** Computed coefficients in the calibration process of the 1500 µm-long, 900 µm-wide cantilevers operating in the second order roof tile-shaped mode. Errors of the calibration method in the determination of the density $\epsilon_{\rho }$ and viscosity $\epsilon_{\eta }$ are also given.

Thickness (µm)	$C_{1}$	$C_{2}$	$C_{3}$	$C_{4}$	$\epsilon_{\rho }\left(\%\right)$	$\epsilon_{\eta }\left(\%\right)$
10	0.1315	1.1671	3.0401	−6.608	5.92	37.46
20	0.2436	2.8588	3.9363	−167.6	6.22	24.68
40	0.1634	3.1960	5.3430	−307.5	1.99	20.25
60	0.2555	11.26	33.07	−4287	7.33	21.73
